# Validation of the FASILA Score for Predicting Interventions and Outcomes in Traumatic Abdominal and Pelvic Injuries: A Prospective Clinical Study

**DOI:** 10.1002/wjs.12632

**Published:** 2025-05-19

**Authors:** Ayman El‐Menyar, Ahmed F. Ramzee, Basel H. Elmegabar, Mohammad Asim, Ruben Peralta, Vishwajit Verma, Husham Abdelrahman, Hisham Jogol, Muniba Afzal, Yasir Abdulrahman, Nuri Abdurraheim, Ahad Kanbar, Tariq Siddiqui, Sandro Rizoli, Hassan Al‐Thani

**Affiliations:** ^1^ Clinical Research, Trauma and Vascular Surgery Hamad Medical Corporation (HMC) Doha Qatar; ^2^ Clinical Medicine Weill Cornell Medical College Doha Qatar; ^3^ Department of Surgery Trauma HMC Doha Qatar; ^4^ Department of Surgery Universidad Nacional Pedro Henriquez Urena Santo Domingo Dominican Republic; ^5^ Department of Surgery General Surgery HMC Doha Qatar

**Keywords:** FASILA score, abdominal trauma, pelvic, Injury, blood transfusion, surgical intervention, and mortality

## Abstract

**Background:**

The FASILA score is a 7‐point scale comprising the FAST (focused assessment with sonography in trauma), shock index, and serum lactate. We aimed to validate this score prospectively in patients with abdominal and pelvic trauma to predict the need for massive blood transfusion, laparotomy, and inhospital mortality.

**Methods:**

This prospective study included all adult patients who sustained abdominal and/or pelvic trauma. Pediatric patients, prehospital cardiac arrest, and severe head injury were excluded. Data were analyzed and compared with low (< 4) versus high (≥ 4) FASILA scores.

**Results:**

A total of 400 patients (mean age 36.3 ± 12.7; 90% male; and 96% had blunt trauma) were enrolled between 2022 and 2024. Patients with higher FASILA scores (19.3%) had higher rates of exploratory laparotomy and radiological interventions and received more blood transfusions. They also had longer hospital stays, higher rates of sepsis, and mortality (*p* = 0.001). The FASILA scores significantly correlated with shock index (*r* = 0.75), ISS (*r* = 0.38), SOFA score (*r* = 0.36), and abdominal AIS (*r* = 0.27), TRISS (*r* = −0.19), RTS (*r* = −0.26), and fibrinogen levels (*r* = −0.12). A FASILA score ≥ 4 had a high specificity (85.5%) and negative predictive value (80%) for predicting the need for surgery. The FASILA score was an independent predictor of blood transfusion (odds ratio 1.92 and 95% CI 1.43–2.58) after adjusting for abdominal AIS, ISS, SOFA score, and fibrinogen level. AUROC curves were better for the FASILA score than ABC score and “SI alone” for the prediction of MTP and surgical interventions.

**Conclusion:**

The significant association between high FASILA scores and blood transfusions, surgical interventions, and mortality indicates its usefulness in early risk stratification in abdominopelvic trauma. Multicenter studies are recommended to enhance external validity across diverse populations and settings.

## Graphical abstract

The FASILA score is a 7‐point scale comprising the FAST (focused assessment with sonography in trauma), shock index, and serum lactate. We aimed to validate this score prospectively in patients with abdominal and pelvic trauma to predict the need for massive blood transfusion, laparotomy, and inhospital mortality. This prospective study included all adult patients who sustained abdominal and/or pelvic trauma. Pediatric patients, prehospital cardiac arrest, and severe head injury were excluded. Data were analyzed and compared with low (< 4) versus high (≥ 4) FASILA scores.

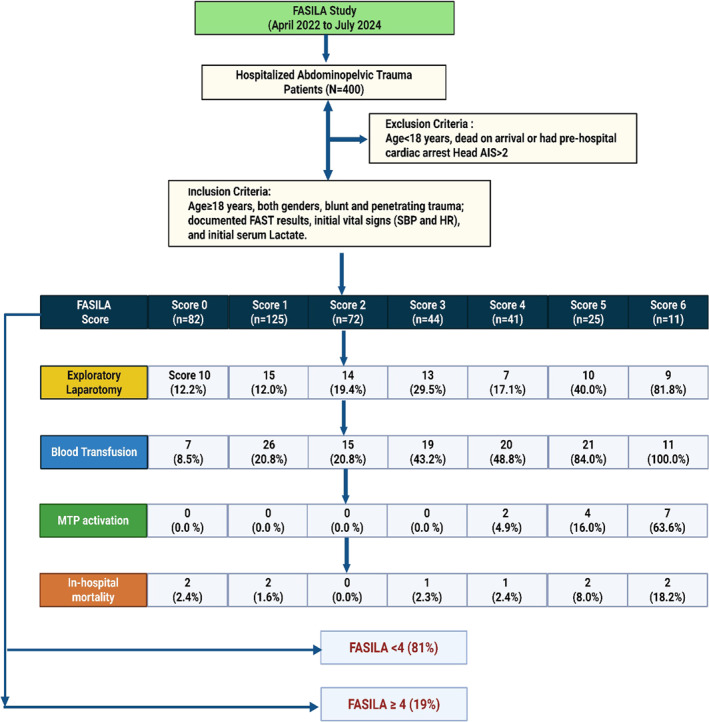

## 1 Introduction

In polytrauma patients, injuries to the abdomen and pelvis are common, often from high‐energy motor vehicle crashes [1]. Abdominal trauma occurs in approximately 1/4 of all traumatic injuries worldwide [2, 3]. In Qatar, abdominal trauma is present in 12.5%–15% of all trauma‐related admissions [4, 5] and 9% have concurrent pelvic injuries [5]. Hemorrhage is the leading preventable cause of trauma‐related mortality, contributing to nearly half of all deaths in the first 24 h postinjury [6, 7].

Many scoring systems are used in trauma, such as the shock index (SI), Assessment of Blood Consumption (ABC), modified Trauma‐Induced Coagulopathy Clinical Score (mTICCS), and clinical abdominal scoring system (CASS). Based on initial hemodynamic and other parameters, these scores are bedside triage tools for rapid identification of patients likely to require massive transfusion and/or exploratory laparotomy [8, 9, 10, 11, 12, 13]. However, some of these scoring systems rely on complex and resource‐intensive methodologies that are not readily available in busy trauma settings [14]. Recent score (BATT), including age, systolic blood pressure (SBP), respiratory rate (RR), Glasgow Coma Scale (GCS), heart rate (HR), penetrating injury, and high‐velocity trauma, aims to predict death due to bleeding. BATT scores ≥ 2 predict better outcomes, and tranexamic acid can reduce bleeding‐related death by 4 times [15]. To predict the need for activation of the massive transfusion protocol (MTP), ABC score should be ≥ 2 (1 point for each of: penetrating injury, positive FAST, SBP ≤ 90, and HR ≥ 120) [11]. However, studies showed that SI is more sensitive and requires fewer skills than the ABC score [16, 17, 18].

The FASILA score is a novel bioclinical scoring tool that integrates clinical parameters readily available during trauma resuscitation, including FAST (focused assessment with sonography in trauma), SI, and initial serum lactate [16]. We recently found the FASILA score to be a fast, simple, and accurate predictor of emergency interventions and mortality [19]. The FASILA score correlates with existing scoring systems, outperforming the widely used ABC and SI scores in predicting massive transfusion, exploratory laparotomy, and mortality in patients with abdominal trauma [16, 20]. This could lead to more effective resuscitation and ultimately improve patient outcomes. FASILA was developed using retrospective data of patients with any abdominal trauma. It has only been validated in small prospective observational cohorts of patients with abdominal trauma [20], which is relevant for its broader acceptance in clinical practice, assisting in timely decision‐making. Thus, the present study aims to validate the FASILA score prospectively in a large cohort of patients with abdomen and pelvic trauma.

## 2 Methods

This prospective observational study included all patients with abdominal and/or pelvic trauma as determined by the admitting trauma surgeon, admitted to the Hamad Trauma Center (HTC), with documented FAST, initial vital signs (SBP and HR), and serum lactate. Patients under 18 years of age and those declared dead on arrival or who suffered a prehospital cardiac arrest were excluded. Patients with severe head injury (head AIS > 2) would be excluded from the analysis. The HTC manages around 2500 trauma patients annually, with approximately 1500–2000 requiring hospital admission. Of these, 85%–90% are blunt trauma, 45% sustained road traffic injuries (RTIs), and around 15% of trauma admissions are due to abdominal injuries [5, 16, 21]. Based on these estimates, the target sample size for the study was 400 adult abdominal and or patients with pelvic trauma admitted between April 2022 and July 2024. Inclusion criteria included patients (male or female) of age > 17 years old who sustained abdominal and/or pelvic injury (blunt or penetrating) with documented FAST results and serum lactate.

FASILA score, which was developed specifically for patients with abdominal and pelvic injuries, has become an integral standard of practice at HTC for such patients, alongside other scores such as the injury severity score (ISS), abbreviated injury score (AIS), and GCS. The FASILA score is calculated by adding the following components: FAST test results (negative = 0 and positive = 1), SI values (0 for 0.50–0.69, 1 for 0.70–0.79, 2 for 0.80–0.89, and 3 for ≥ 0.90), and initial serum lactate levels (0 for ≤ 2.0 mmol/L, 1 for 2.0–4.0 mmol/L, and 2 for ≥ 4.0 mmol/L) [16]. The FASILA score ranges from 0 to 6. The serum lactate cutoff in the FASILA score was determined based on evidence/publications and well‐established thresholds [22, 23]. We have developed a mobile application that enables our trauma team to calculate and document easily. The in‐charge physician and nursing staff independently enter the four value components of the FASILA score simultaneously, which are “the pulse rate and SBP for the shock index [SI], FAST scan result, and serum lactate value” into the mobile App to get instant and accurate results. The mobile application is easily and exclusively downloaded by the trauma team physicians and nurses who are trained to use it. These data are also available on the hospital’s electronic medical record (EMR).

Data from the National Trauma Registry database and additional relevant variables were retrieved as well, such as demographics (age and sex), type of trauma (blunt or penetrating), initial vital signs (SBP, diastolic blood pressure, and HR) at emergency department (ED), diastolic SI (pulse rate/diastolic blood pressure) and systolic SI (pulse rate/SBP) at ED, abdominal abbreviated injury score, initial fibrinogen levels, ISS, GCS at ED, revised trauma score (RTS), trauma score and injury severity score (TRISS), sequential organ failure assessment score (SOFA), exploratory laparotomy, intubation in emergency department, and management such as conservative, surgical intervention, intervention radiology (IR), conservative plus IR, and surgical intervention plus IR. We also collected data for vasopressors used, need for blood transfusion, number of blood units transfused, MTP activation, length of intensive care unit (ICU) and hospital stay, in‐hospital complications (sepsis and acute kidney injury), and mortality.

The SOFA score is a tool used to assess the function of multiorgan systems, including neurological, hematologic, hepatic, renal, and hemodynamic systems, by assigning a score based on specific data collected for each category, with a range of 0–24 [24]. Massive transfusion is defined as the administration of 10 or more units of packed red blood cells (PRBCs) within 24 h of hospital admission. Serum lactate was measured on arrival using POC testing (ABL90 FLEX blood gas analyzer).

The primary outcome measure was to validate the FASILA score for its utility in guiding clinical decision‐making for the need for surgical intervention and outcomes. The study adhered to the institution’s ethical standards and was approved by the Institutional Research Board of the Medical Research Center, Hamad Medical Corporation (IRB # MRC‐01‐21‐523). Consent was waived since there was no direct interaction with patients, and data analysis was conducted anonymously.

### 2.1 Statistical Analysis

Data were presented as means ± standard deviations (SDs) and medians (interquartile range) as appropriate for continuous variables and as frequencies and proportions for categorical variables. The preset sample size was not calculated, as we intended to recruit all eligible patients within the study period. Post hoc analysis was performed using the two‐proportion *z* test to assess the study power. The FASILA scores were divided into seven levels, ranging from 0 to 6, and these 7‐point FASILA scales were analyzed and compared. Furthermore, the FASILA scores were grouped into two categories: low (< 4) and high (≥ 4) and the differences between the groups were analyzed. The FASILA score cutoff of 4 was adopted as per the previous publications [16, 20]. Categorical variables were analyzed using the chi‐squared or Fisher's exact test. Continuous variables were analyzed using either the student's *t* or analysis of variance (ANOVA) test. Multivariable logistic regression analysis was performed for predictors of blood transfusion using relevant and significant variables on the univariate analysis. Data were expressed as odds ratio (OR) and 95% confidence intervals (CIs).

A receiver operating characteristic (ROC) curve analysis was performed on the different scoring systems to predict the need for surgical intervention. The area under the ROC curve (AUROC) was calculated to evaluate the performance and discriminatory power of the FASILA score ≥ 4, systolic SI > 0.80, and diastolic SI > 2.2. These scoring systems’ sensitivity, specificity, positive predictive value (PPV), and negative predictive value (NPV) in predicting the need for surgical intervention were determined. Moreover, as a validation, AUROC curves were used to compare the FASILA and ABC scores as predictors for MTP and surgical interventions. Correlation coefficients were used to measure the strength of the relationship between the FASILA score and SI, RTS, TRISS, ISS, serum lactate, SOFA score, blood units transfused, ICU length of stay, fibrinogen levels, and abdominal AIS. A two‐sided *p* < 0.05 was considered statistically significant. All statistical analyses were performed using Statistical Package for the Social Sciences (SPSS) for Windows version 21.0 (SPSS Inc.; Chicago, IL, USA).

## 3 Results

### 3.1 Overall

**FIGURE 1:**
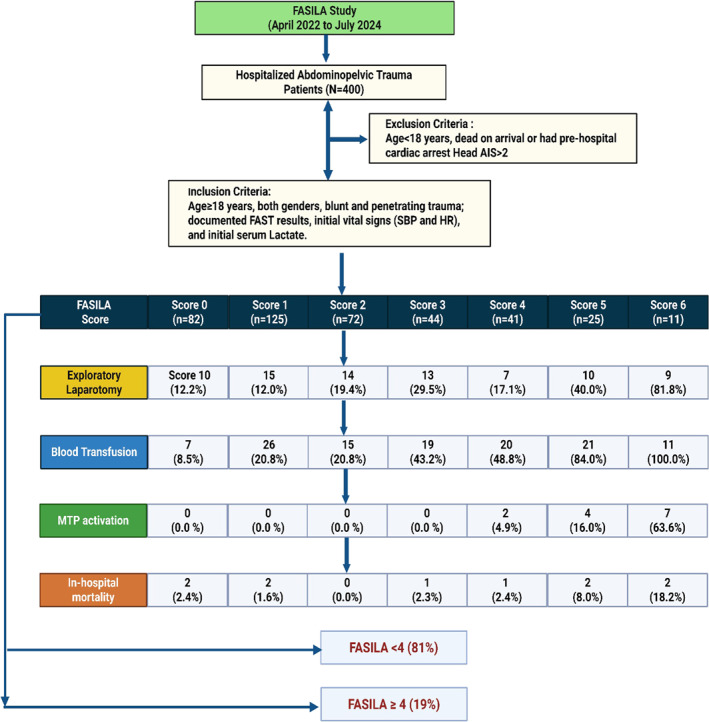
Study design.

As planned, 400 patients with abdominal and pelvic trauma were prospectively included in the present study (Figure 1). The mean age of patients was 36.3 ± 12.7; the majority were males (90.3%), and 96% had blunt trauma. Two‐thirds (67.7%) were managed conservatively, 23.5% required surgical interventions, and 6.5% underwent IR (Tables 1 and 2). Overall, 119 (29.8%) patients received blood transfusions, only 3.3% required MTP activation, and the overall mortality rate was 2.5%.

**TABLE 1:** Patient's characteristics, laboratory findings, injury scores, and hospital course based on the 7‐point FASILA score (*n* = 400).

Variable	Score 0 (*n* = 82)	Score 1 (*n* = 125)	Score 2 (*n* = 72)	Score 3 (*n* = 44)	Score 4 (*n* = 41)	Score 5 (*n* = 25)	Score 6 (*n* = 11)	*p* value
Age (mean ± SD) years	38.0 ± 15.3	37.3 ± 12.4	34.7 ± 10.2	32.9 ± 12.7	36.3 ± 11.3	32.8 ± 10.5	44.7 ± 16.1	0.03
Males	74 (90.2%)	110 (88.0%)	68 (94.4%)	39 (88.6%)	38 (92.7%)	23 (92.0%)	9 (81.8%)	0.73
GCS ED	15 (3–15)	15 (3–15)	15 (3–15)	15 (3–15)	15 (3–15)	15 (3–15)	13 (3–15)	0.001
Shock index ED	0.60 ± 0.08	0.63 ± 0.10	0.74 ± 0.09	0.86 ± 0.19	1.07 ± 0.29	1.24 ± 0.36	1.25 ± 0.28	0.001
Abdominal AIS	2.45 ± 0.60	2.51 ± 1.21	2.63 ± 0.87	2.97 ± 1.47	2.94 ± 0.72	2.91 ± 1.06	4.09 ± 0.83	0.001
RTS	7.73 ± 0.56	7.79 ± 0.35	7.72 ± 0.65	7.52 ± 0.96	7.55 ± 0.72	7.17 ± 1.64	6.10 ± 2.27	0.001
TRISS	0.98 ± 0.07	0.98 ± 0.03	0.98 ± 0.04	0.94 ± 0.16	0.95 ± 0.12	1.00 ± 0.17	0.70 ± 0.38	0.001
ISS (median and IQR)	9 (5–17)	14 (9–17)	14 (9–17)	17 (9–22)	19 (13–26)	22 (14–28)	34 (17–39)	0.001
SOFA score	1 (0–9)	1 (0–5)	1 (0–5)	2 (0–14)	2.5 (0–12)	2.5 (0–10)	3 (1–15)	0.001
Exploratory laparotomy	10 (12.2%)	15 (12.0%)	14 (19.4%)	13 (29.5%)	7 (17.1%)	10 (40.0%)	9 (81.8%)	0.001
Intubation in TRU	0 (0.0%)	4 (3.2%)	1 (1.4%)	1 (2.3%)	4 (9.8%)	2 (8.0%)	1 (9.1%)	0.05
Management								
Conservative only	68 (82.9%)	98 (78.4%)	48 (66.7%)	25 (56.8%)	22 (53.7%)	9 (36.0%)	1 (9.1%)	0.001 for all
Surgical intervention	9 (11.0%)	19 (15.2%)	21 (29.2%)	15 (34.1%)	13 (31.7%)	10 (40.0%)	7 (63.6%)	
Intervention radiology	3 (3.7%)	6 (4.8%)	3 (4.2%)	4 (9.1%)	4 (9.8%)	5 (20.0%)	1 (9.1%)	
Conservative plus IR	2 (2.4%)	2 (1.6%)	0 (0.0%)	0 (0.0%)	2 (4.9%)	0 (0.0%)	0 (0.0%)	
Surgical intervention plus IR	0 (0.0%)	0 (0.0%)	0 (0.0%)	0 (0.0%)	0 (0.0%)	1 (4.0%)	2 (18.2%)	
ICU LOS	2 (1–19)	2 (1–25)	2 (1–13)	4 (1–41)	4 (1–124)	4 (1–23)	6 (1–38)	0.001
HLOS	5 (1–62)	6 (1–72)	6 (1–76)	10 (1–107)	18 (1–171)	16 (1–109)	18 (3–97)	0.001
Ventilatory days	2.5 (1–15)	1 (1–9)	1 (1–4)	6 (1–42)	3 (1–66)	2 (1–23)	4 (1–22)	0.01
Blood transfusion	7 (8.5%)	26 (20.8%)	15 (20.8%)	19 (43.2%)	20 (48.8%)	21 (84.0%)	11 (100%)	0.001
Number of blood units	5 (3–9)	2 (1–11)	4 (2–16)	3 (1–14)	4 (1–84)	4 (1–28)	18 (1–47)	0.002
MTP activation	0 (0.0%)	0 (0.0%)	0 (0.0%)	0 (0.0%)	2 (4.9%)	4 (16.0%)	7 (63.6%)	0.001
Inhospital mortality	2 (2.4%)	2 (1.6%)	0 (0.0%)	1 (2.3%)	1 (2.4%)	2 (8.0%)	2 (18.2%)	0.01

**TABLE 2:** Patient’s characteristics, injury scores, management, and hospital course based on low versus high FASILA scores.

Variable	FASILA score < 4 (*n* = 323; 80.8%)	FASILA score ≥ 4 (*n* = 77; 19.3%)	*p* value
Age (mean ± SD) years	36.3 ± 12.8	36.4 ± 12.3	0.99
Males	291 (90.1%)	70 (90.9%)	0.82
Blunt trauma	309 (95.7%)	75 (97.4%)	0.48 for all
Penetrating trauma	14 (4.3%)	2 (2.6%)	
Diastolic shock index ED	1.10 ± 0.23	1.70 ± 0.48	0.001
Systolic shock index ED	0.67 ± 0.14	1.16 ± 0.32	0.001
Abdominal AIS	2.6 ± 1.1	3.1 ± 0.9	0.001
Pelvic AIS	2.4 ± 0.8	2.5 ± 0.9	0.55
Revised trauma score (RTS)	7.7 ± 0.6	7.2 ± 1.4	0.001
TRISS	0.97 ± 0.08	0.93 ± 0.20	0.006
Injury severity score^a^	13 (8–17).	22 (14–27)	0.001
SOFA score	1 (0–14)	3 (0–15)	0.001
Fibrinogen level < 2.0 g/L	37 (15.1%)	18 (27.3%)	0.02
Exploratory laparotomy	52 (16.1%)	26 (33.8%)	0.001
Intubation in TRU	6 (1.9%)	7 (9.1%)	0.001
Management			
Conservative only	239 (74.0%)	32 (41.6%)	0.001 for all
Conservative plus IR	4 (1.2%)	2 (2.6%)	
Surgical intervention	64 (19.8%)	30 (39.0%)	
IR only	16 (5.0%)	10 (13.0%)	
Surgical intervention plus IR	0 (0%)	3 (3.9%)	
Hospital LOS	7 (1–107)	17.5 (1–171)	0.001
ICU LOS	2 (1–41)	4 (1–124)	0.001
Blood transfusion	67 (20.7%)	52 (67.5%)	0.001
Blood units transfused	3 (1–16)	5 (1–84)	0.002
MTP activation	0 (0.0%)	13 (16.9%)	0.001
Inotropes used	6 (1.9%)	17 (22.1%)	0.001
Inhospital complications			
Sepsis	0 (0.0%)	2 (2.6%)	0.04
Acute kidney injury	0 (0.0%)	3 (3.9%)	0.004
Inhospital mortality	5 (1.5%)	5 (6.5%)	0.01

Abbreviations: IR, intervention radiology and LOS, length of stay.

^a^ median and interquartile range.

The 7‐point FASILA score analysis (Table 1): The need for blood transfusion (*p* = 0.001), MTP (*p* = 0.001), and a median number of blood units transfused (*p* = 0.002) were significantly higher in patients with the highest FASILA scores (Table 1). Patients with higher scores required longer mechanical ventilation (*p* = 0.01), ICU (*p* = 0.001), and hospital stays (*p* = 0.001) and had a higher rate of inhospital mortality (*p* = 0.01) compared to the other groups.

Low versus high FASILA score groups: Table 2 compares clinical characteristics, management, and outcomes based on low versus high FASILA scores. The two groups were comparable in age, sex, and mechanism of injury. The ISS (*p* = 0.001), abdominal AIS (*p* = 0.001), diastolic SI (*p* = 0.001), systolic SI (*p* = 0.001), and SOFA scores (*p* = 0.001) were significantly higher in patients with a FASILA score ≥ 4. Hypofibrinogenemia (*p* = 0.02) and exploratory laparotomy (*p* = 0.001) were also more frequent in the group with high FASILA scores.

**FIGURE 2:**
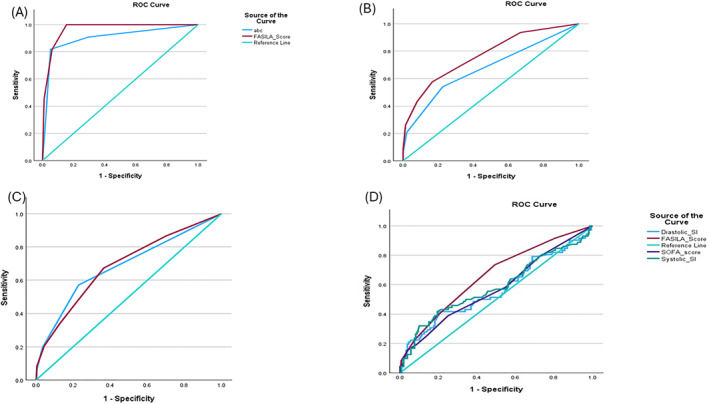
Area under the ROC curves comparing FASILA and other scoring tools: (A) For prediction of massive blood transfusion protocol activation: Area under the ROC (AUROC) curve: FASILA score 0.96 and ABC score 0.90. (B) For any blood transfusion: AURCO curve: FASILA score 0.77 and ABC score 0.67. (C) For surgical intervention including exploratory laparotomy: AUROC curve: FASILA score 0.69 and ABC score 0.68 (D) Discriminatory power analysis for surgical intervention showing the area under the curve for the systolic shock index 0.58; FASILA score 0.66; diastolic shock index 0.57; and SOFA score 0.57.

Correlations, discriminatory power, and post hoc analyses: Table 3 shows the significant correlations between the FASILA score and injury severity indicators, laboratory findings, blood transfusion, and ICU stay duration. Table 4 and Figure 2 show the discriminatory analysis of different scoring systems to predict the need for surgical intervention. FASILA scores outperformed systolic and diastolic SI and SOFA scores. Post hoc power analysis showed that the test priori power is a strong predictor of blood transfusion compared to the two study groups (97.63%).

**TABLE 3:** Correlation analysis between FASILA score and injury severity indicators, laboratory findings, blood transfusion, and intensive care unit sta**y**.

Systolic shock index	Pearson correlation	0.751^a^
	Sig. (2‐tailed)	0.001
	*N*	400
Revised trauma score	Pearson correlation	−0.260^a^
	Sig. (2‐tailed)	0.001
	*N*	392
Trauma injury severity score	Pearson correlation	−0.192^a^
	Sig. (2‐tailed)	0.001
	*N*	391
Injury severity score	Pearson correlation	0.384^a^
	Sig. (2‐tailed)	0.001
	*N*	399
Serum lactate	Pearson correlation	0.093^b^
	Sig. (2‐tailed)	0.063
	*N*	398
SOFA score	Pearson correlation	0.367^a^
	Sig. (2‐tailed)	0.001
	*N*	255
Blood units transfused	Pearson correlation	0.310^a^
	Sig. (2‐tailed)	0.001
	*N*	119
ICU LOS	Pearson correlation	0.253^a^
	Sig. (2‐tailed)	0.001
	*N*	213
Fibrinogen level	Pearson correlation	−0.127^a^
	Sig. (2‐tailed)	0.025
	*N*	309
Abdomen AIS	Pearson correlation	0.272^a^
	Sig. (2‐tailed)	0.001
	*N*	287

^a^ Correlation is significant at the 0.01 level.

^b^ Correlation is significant at the 0.05 level (2‐tailed).

**TABLE 4:** Discriminatory power analysis of different scoring systems to predict the need for surgical intervention.

	The area under the curve	Sensitivity (95% CI)	Specificity (95% CI)	PPV (95% CI)	NPV (95% CI)
FASILA ≥ 4	0.7	34.0% (24.7%–44.3%)	85.5% (81.0%–89.3%)	42.8% (33.7%–52.5%)	80.2% (77.7%–82.5%)
Systolic SI > 0.80	0.6	43.3% (33.3%–53.8%)	74.6% (69.3%–79.4%)	35.3% (28.8%–42.4%)	80.4% (77.3%–83.2%)
Diastolic SI > 2.2	0.6	7.2% (2.9%–14.3%)	98.7% (96.6%–99.6%)	63.6% (34.4%–85.4%)	76.9% (75.8%–77.9%)

Validation of the FASILA score compared to other triage score tool: Figure 2 shows comparisons of AUROC curves between FASILA and ABC scores regarding the prediction of MTP and surgical interventions including exploratory laparotomy. It also shows a comparison between FASILA and systolic SI, diastolic SI, and SOFA scoring tools.

**FIGURE 3:**
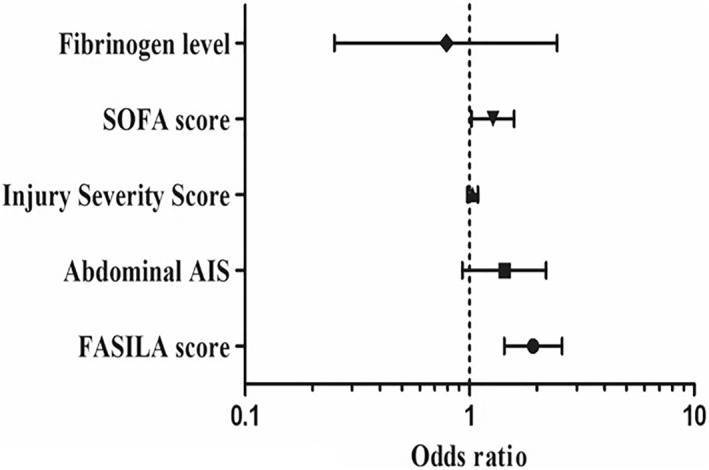
Predictors of blood transfusion in abdominopelvic injuries.

Multivariable logistic regression analysis: multivariable analysis demonstrated that a FASILA score > 4 is an independent predictor of blood transfusion (odds ratio 1.92 and 95% CI 1.43–2.58) after adjusting for an abdominal AIS, ISS, Sofa score, and fibrinogen level (Figure 3). Adjusted variables did not include SI, serum lactate, and FAST, as they are already components of the FASILA scoring system.

## 4 Discussion

The present study prospectively validated the utility of the FASILA score in a large cohort of patients. It demonstrates its reliability as a readily obtainable prognostic tool for MTP, exploratory laparotomy, and mortality in patients with abdominal and/or pelvic trauma. The study also demonstrated a significant correlation with other scoring systems, positively with ISS and SOFA and negatively with TRISS and fibrinogen levels.

In brief, a high FASILA score (≥ 4) represented one‐fifth of all patients abdominopelvic trauma and was associated with greater injury severity and higher rates of interventions, hospital stay, complications, and mortality as well as a 2‐fold increase in blood transfusions.

During initial resuscitation, in the noise/chaos trauma room, information is often lacking and incomplete, many diagnostic investigations are not done, and the clinician must rely on “intuition” to determine whether to escalate care or not—and mistakes in this phase of resuscitation are unfortunately common. Individual prognostic parameters always have shortcomings. Scores, such as FASILA (on mobile app and EMR), could alert clinicians to injury severity and the likelihood of needing blood and surgery, thus making the correct decisions in a few minutes. The use of SI increases the sensitivity of SBP as well as the specificity of HR; adding simple physiological parameters that suggest bleeding, such as the point of care ultrasound (FAST) and lactate, would help the clinical decision in recognizing shock state and initiating “stop the blood” process on time. A recent study by Rizk et al. [20] demonstrated a significant positive correlation between the FASILA score, the ABC score, and SI, highlighting its potential for practical integration into existing trauma protocols. Notably, after adjusting for potential confounders, they identified the FASILA score as the independent predictor of MTP [20]. The present study, using area under the ROC curves, compared the discriminatory power of FASILA and other triage scoring tools. AUROC curves were better for the FASILA score than ABC score and “SI alone” for the prediction of MTP and surgical interventions.

Furthermore, the FASILA score was found to have higher discriminatory power than established scoring systems, such as ABC, RABT, and SI, in patients with abdominal trauma [16, 25]. This highlights its robustness and potential to provide a holistic assessment of trauma severity, enables appropriate triage and clinical decision‐making, and outperforms traditional scoring systems in patients with abdominal trauma [20].

An earlier study by Škola et al. [26] highlighted that early high SI has moderate sensitivity, high specificity, and an excellent negative predictive value for predicting hypofibrinogenemia. Supporting this, our findings suggest that the FASILA score, which incorporates SI as a key component, can effectively detect trauma patients who are at risk of early hypofibrinogenemia necessitating early management [27].

Moreover, our study demonstrated that a FASILA score ≥ 4 was associated with doubling ICU and hospital stays. Of note, a FASILA score, with its high specificity and negative predictive value, can be a reliable triage tool to identify patients less likely to require surgery. Thus, unnecessary procedures could be reduced and resource allocation optimized. This potential is relevant in any resource‐limited settings or during mass casualty events, where quick and accurate decision‐making is crucial.

### 4.1 Limitations

This prospective study has some limitations. First, its single‐center design may limit the generalizability of the findings to other healthcare settings, and this also reflects a single‐center validation bias. However, we compared FASILA and another triage scoring tools to improve the validation of the present score. Second, FASILA, as a relatively new scoring system, lacks external validation in different populations. Validating the score by the same team that developed the score may be a source of bias. However, the current validation was carried out as a prospective study a few years after the retrospective one, and this score has been validated recently by another group in another country [20]. There are potential biases, particularly for subgroups, such as pediatric and geriatric patients, as well as the general trauma population, expanding beyond abdominopelvic injuries only. Factors that could alter the initial SI [28, 29, 30, 31] and inconclusive FAST results need to be considered and elaborated more. Most patients were males and only 10% were females. This may lead to sex‐biased results; however, it reflects the nature of trauma in the country [21, 32].

## 5 Conclusion

The association between high FASILA scores and blood transfusion, surgical interventions, and mortality indicates its usefulness in early risk stratification. Moreover, the FASILA score components are easily obtainable clinical parameters. FASILA score facilitates rapid decision making for appropriate on‐time intervention in abdominopelvic trauma. Further, multicenter research with a more diverse population would strengthen its validity.

## Author Contributions

**Ayman El‐Menyar:** conceptualization, investigation, writing – original draft. **Ahmed F. Ramzee:** data curation, methodology. **Basel H. Elmegabar:** data curation. **Mohammad Asim:** data curation, formal analysis. **Ruben Peralta:** supervision, investigation. **Vishwajit Verma:** software, investigation. **Husham Abdelrahman:** writing – review and editing, conceptualization. **Hisham Jogol:** data curation, methodology. **Muniba Afzal:** data curation. **Yasir Abdulrahman:** investigation. **Nuri Abdurraheim:** data curation. **Ahad Kanbar:** data curation. **Tariq Siddiqui:** data curation. **Sandro Rizoli:** writing – review and editing, supervision. **Hassan Al‐Thani:** supervision, methodology.

## Ethics Statement

Ethical approval was obtained from the institutional review board (MRC‐01‐21‐523) at the Medical Research Center, Hamad Medical Corporation (HMC), Doha, Qatar.

## Consent

The authors have nothing to report.

## Conflicts of Interest

The authors declare no conflicts of interest.

## Data Availability

All data are presented in the manuscript. After a reasonable research request and signed data share agreement, further access needs approval from the medical research center at Hamad Medical Corporation.
